# Supplementary material for: The interactome of intact mitochondria by cross-linking mass spectrometry provides evidence for coexisting respiratory supercomplexes
Source: Mol Cell Proteomics. 2017 Dec 8;17(2):216–32. doi: 10.1074/mcp.RA117.000470 (PMC5795388; doi:10.1074/mcp.RA117.000470)
Supplement: Supplemental Data [file supp_RA117.000470_133922_0_supp_23978_fzffwf.zip › spectra_annotation/mito_DR_spectra_annotation/427-1-1-1-57-1.pdf]

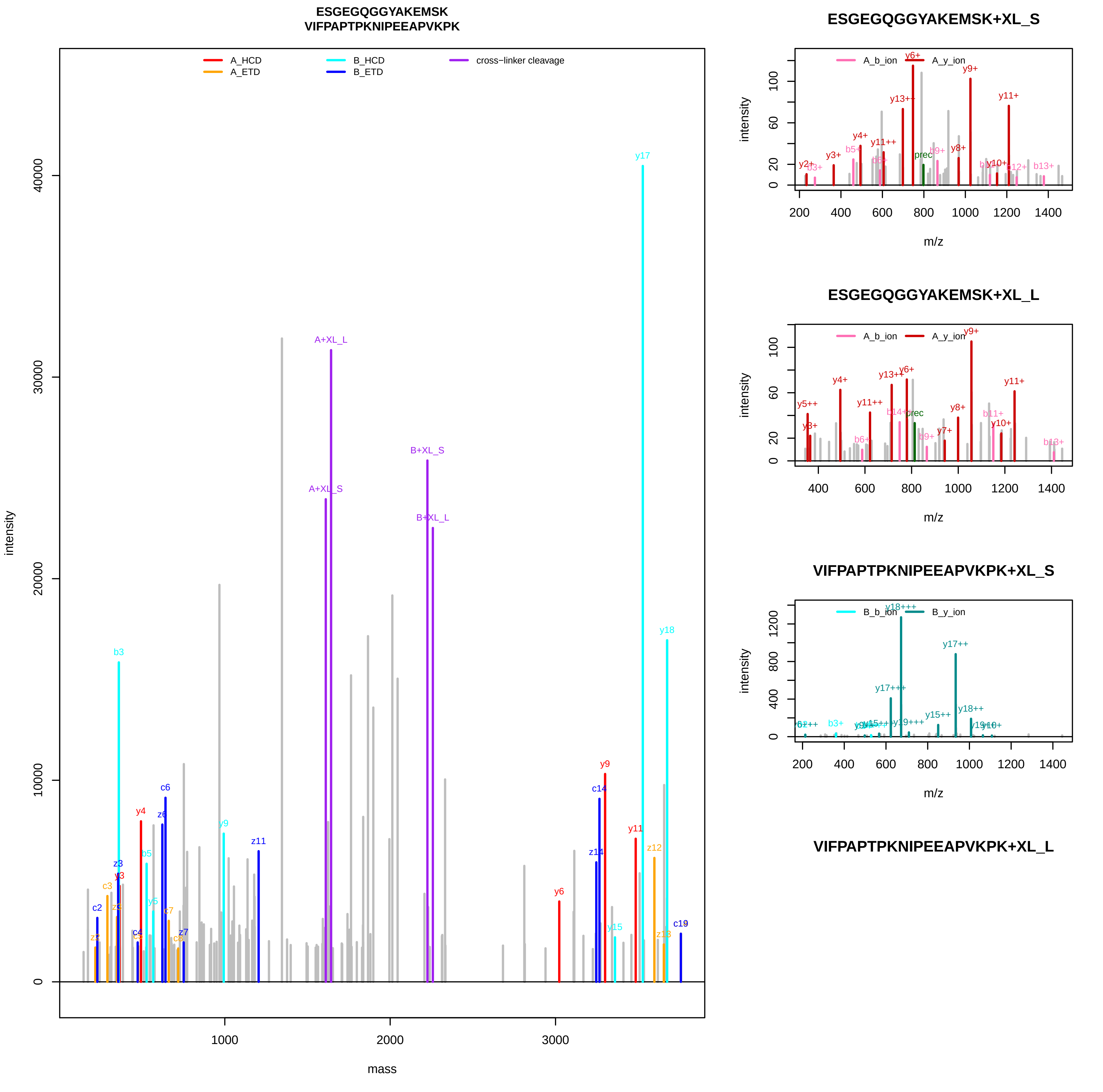

ESGEGQGGYAKEMSK
VIFPAPTPKNIPEEAPVKPK
A_HCD
A_ETD
B_HCD
B_ETD
cross−linker cleavage
intensity
mass
0
10000
20000
30000
40000
1000
2000
3000
y17
A+XL_L
B+XL_S
A+XL_S
B+XL_L
b3
y18
y9
y11
c14
z14
c6
z6
y4
z11
y6
z12
c19
ESGEGQGGYAKEMSK+XL_S
A_b_ion
A_y_ion
intensity
m/z
y6+
y9+
y11+
y13++
y4+
y11++
y3+
y2+
b3+
b5+
b6+
prec
b9+
y8+
y10+
b12+
b13+
ESGEGQGGYAKEMSK+XL_L
y9+
y4+
y13++
y6+
y11+
y11++
y5++
y3+
b6+
b14+
prec
b9+
y7+
y8+
b11+
y10+
b13+
VIFPAPTPKNIPEEAPVKPK+XL_S
B_b_ion
B_y_ion
y18+++
y17++
y17+++
y18++
y15++
y19+++
b3+
VIFPAPTPKNIPEEAPVKPK+XL_L
